# The Aurora B Kinase in Chromosome Bi-Orientation and Spindle Checkpoint Signaling

**DOI:** 10.3389/fonc.2015.00225

**Published:** 2015-10-16

**Authors:** Veronica Krenn, Andrea Musacchio

**Affiliations:** ^1^Department of Mechanistic Cell Biology, Max Planck Institute of Molecular Physiology, Dortmund, Germany; ^2^Faculty of Biology, Centre for Medical Biotechnology, University Duisburg-Essen, Essen, Germany

**Keywords:** centromere, kinetochore, spindle assembly checkpoint, kinase, phosphatase, Aurora B, chromosome passenger complex, bi-orientation

## Abstract

Aurora B, a member of the Aurora family of serine/threonine protein kinases, is a key player in chromosome segregation. As part of a macromolecular complex known as the chromosome passenger complex, Aurora B concentrates early during mitosis in the proximity of centromeres and kinetochores, the sites of attachment of chromosomes to spindle microtubules. There, it contributes to a number of processes that impart fidelity to cell division, including kinetochore stabilization, kinetochore–microtubule attachment, and the regulation of a surveillance mechanism named the spindle assembly checkpoint. In the regulation of these processes, Aurora B is the fulcrum of a remarkably complex network of interactions that feed back on its localization and activation state. In this review, we discuss the multiple roles of Aurora B during mitosis, focusing in particular on its role at centromeres and kinetochores. Many details of the network of interactions at these locations remain poorly understood, and we focus here on several crucial outstanding questions.

## General Remarks

**Figure 1:**
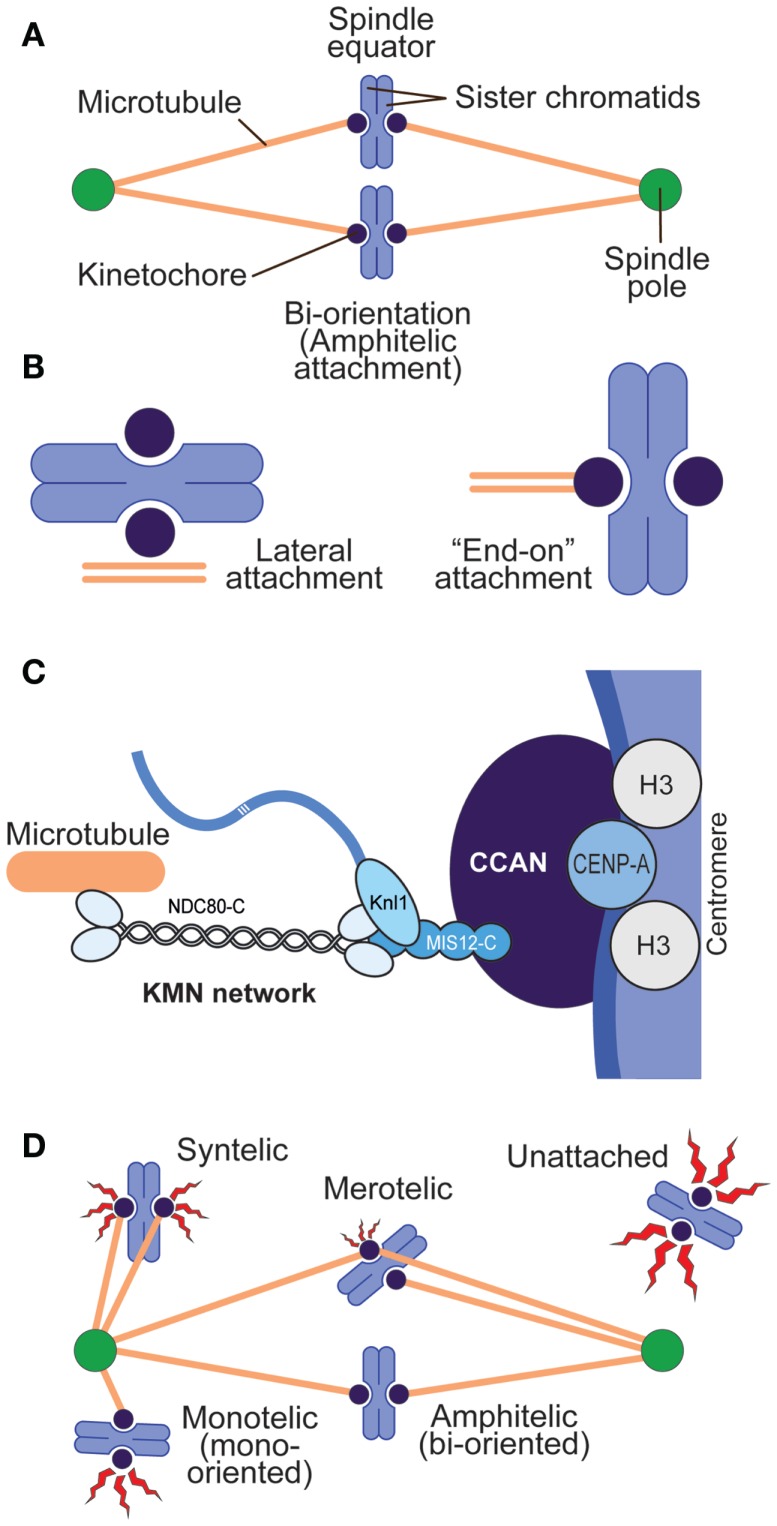
**Chromosome–spindle interactions**. **(A)** A simple spindle with two chromosomes at metaphase. When chromosomes are bi-oriented, the sister chromatids are attached to microtubules, and the microtubules point to opposite spindle poles. **(B)** Two main modes of kinetochore–microtubule attachment predominate in mitosis. Lateral attachment (left) to the microtubule lattice (as opposed to the microtubule end) is typical of early phases of chromosome congression to the equatorial plane of the mitotic spindle and may not fully engage the core kinetochore machinery devoted to microtubule binding but rather molecular motors (7). “End-on” attachment (right) is typical of the final stages of attachment and involves core kinetochore machinery. **(C)** Schematic depiction of centromeres and kinetochores. Centromeres host CENP-A, the histone H3 variant, at much higher levels than other segments of the chromosome. CENP-A binds to a subset of 16 or 17 CCAN subunits, collectively represented as a blue oval. The KMN network binds directly to microtubules. **(D)** Various types of kinetochore–microtubule attachment modes, including erroneous attachments that require correction and that will engage the spindle assembly checkpoint (red flashes). Different “offenses” may provide a graded checkpoint response (variable size of the red flash), with lack of attachment providing a more robust response and merotelic attachment a weak one.

Cells executing mitosis are challenged in ways that can jeopardize their viability and survival (1). The duplicated chromosome pairs (sister chromatids) in the mother cell need to orient on the mitotic spindle so that they can be equally distributed to the two daughter cells after the cohesion that holds them together is dissolved at the metaphase-to-anaphase transition. This process of “bi-orientation” requires that the sister chromatids establish stable “end-on” interactions with microtubules emanating from opposite poles of the mitotic spindle (Figures 1A,B) (2–4). Sister chromatids that fail to bi-orient are mis-segregated into the wrong daughter cell, or separated from the bulk of correctly segregated chromosomes forming the primary nucleus of daughter cells and secluded into extra-nuclear structures called micronuclei. Either fate of mis-oriented chromosomes can have dire consequences for cell physiology (5, 6).

Aurora B is a member of the Aurora family of Serine/Threonine (S/T) protein kinases. Originally discovered as a gene required for maintenance of ploidy in *Saccharomyces cerevisiae* and named increase in ploidy-1 (*IPL1*) (8), Aurora B was later found to control several aspects of chromosome segregation in all eukaryotes (9–11). Two additional members of the Aurora family named Aurora A and Aurora C exist in mammals (12, 13). Substrates of these Aurora kinases usually conform to the consensus [RK]-[RK]-X-[TS]-Θ, where X is any residue and Θ is a hydrophobic or aromatic residue. For instance, Aurora B phosphorylates human KNL1 (CASC5) on the RRVSF motif, which, in the non-phosphorylated version, is a recruitment motif for protein phosphatase 1 (PP1) (14–17). Broad analyses of Aurora B substrates and consensus target sequences have been reported (18, 19). Although Aurora kinases share a similar consensus, distinct subcellular localizations ensure that they deliver activity to distinct substrates and regulate different aspects of mitosis (19, 20).

In this review, we discuss the role of Aurora B in the regulation of chromosome segregation, focusing in particular on the roles of Aurora B during prometaphase, the phase of mitosis during which chromosomes attempt to create stable interactions with spindle microtubules. Readers are also referred to comprehensive reviews that discuss the role of Aurora B also in other phases of mitosis (20, 21).

## Introductory Concepts I: Centromeres and Kinetochores

Sister chromatids interact with spindle microtubules through specialized and structurally complex protein assemblies known as kinetochores (22, 23). On each chromosome, the kinetochore is established on a unique genetic locus named the centromere (Figure 1C). Centromeres, which may consist of several million base pairs of DNA in metazoans, are specialized chromatin domains whose hallmark is the enrichment of the histone H3 variant CENP-A (also known as CenH3) (24). At centromeres, CENP-A containing nucleosomes are embedded in histone H3-containing chromatin at a ratio that, even if estimated to be as little as 1 CENP-A nucleosomes over 25 H3 nucleosomes, is greatly superior to that in bulk chromatin (25).

CENP-A acts as a platform for the recruitment of kinetochore proteins collectively defined as the constitutive centromere-associated network (CCAN), most of which localize at centromeres during the entire cell cycle (26). These proteins form the so-called “inner kinetochore.” Upon entry into mitosis, an additional protein complex, the Knl1 complex–Mis12 complex–Ndc80 complex (KMN) network, is recruited to the CCAN. The KMN network in the “outer kinetochore” interacts directly with spindle microtubules (27) (Figure 1C).

## Introductory Concepts II: Error Correction and the Spindle Assembly Checkpoint

Two feedback mechanisms control the process of kinetochore–microtubule attachment during mitosis, and Aurora B contributes decisively to both of them. These pathways are named error correction (EC) and spindle assembly checkpoint (SAC, also known as mitotic checkpoint, metaphase checkpoint, or “wait anaphase signal”). Error correction is a “local” mechanism that allows kinetochores selectively to stabilize interactions with microtubules that drive chromosome bi-orientation and to weaken those interactions that do not, such as the erroneous configurations known as syntelic and merotelic attachment (Figure 1D) (28). This description of EC summarizes the interpretation of pioneering chromosome micromanipulation experiments carried out over 45 years ago by Bruce Nicklas (29), but is nothing more than a statement of fact, partly because we are still far from a full molecular comprehension of EC. EC is believed to depend on the ability of the kinetochore–centromere system to detect tension, associated with bi-orientation, or lack of tension, associated with lack of bi-orientation. While tension at the bi-oriented chromatids suppresses error correction, lack of tension may not necessarily require error correction (e.g., when lack of tension is due to lack of attachment), but it will require it when kinetochores that are bound to microtubules fail to build tension (as in the case of syntelic or merotelic attachments, Figure 1D).

The KMN complex captures dynamic microtubules to create load-bearing attachments (30, 31). For error correction to occur, kinetochore (KMN)–microtubule interactions need to be sufficiently dynamic to allow the destabilization of erroneous attachments. Aurora B is a key component of the error correction machinery (32, 33). Aurora B inhibition through small-molecule inhibitors or inhibitory antibodies stabilizes incorrect attachments (32–36). Conversely, Aurora B overexpression causes continuous disruption of KT–MT attachments (37), while Aurora B re-activation allows the selective destabilization of incorrect attachments (38–40). Many of the proteins at the interface with microtubules are Aurora B substrates (41).

**Figure 2:**
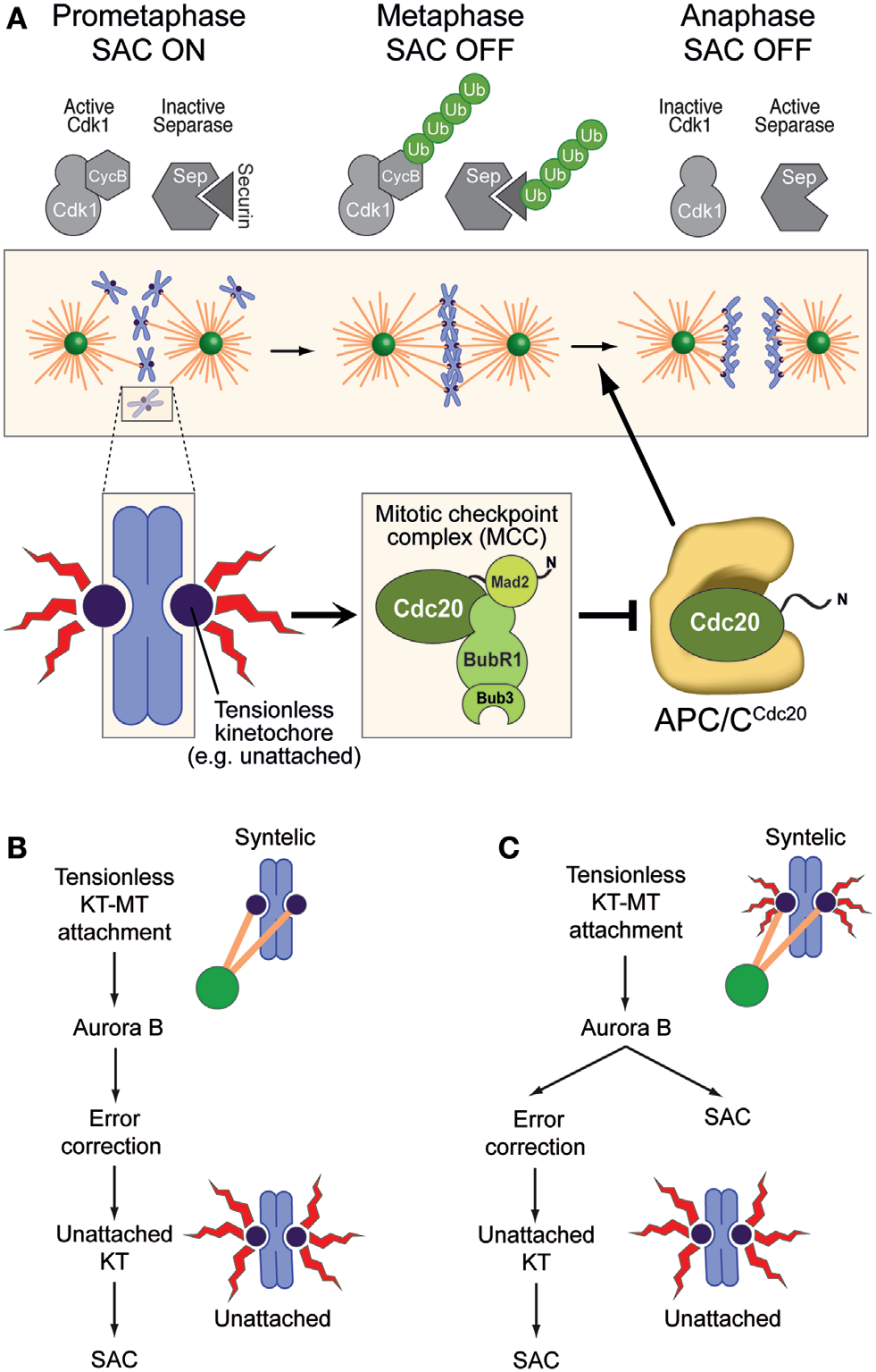
**The spindle assembly checkpoint and error correction**. **(A)** The SAC pathway originates at kinetochores and converges, through several steps, on the assembly of the mitotic checkpoint complex (MCC), which acts as the SAC effector. MCC has been proposed to target the complex of APC/C pre-bound to a second molecule of Cdc20 (which can act both as an APC/C co-activator and as an MCC subunit) (46, 47). APC/C^Cdc20^ promotes poly-ubiquitylation (Ub) of Cyclin B and Securin, promoting mitotic exit and separation of sister chromatids. MCC inhibits this activity of APC/C^Cdc20^ until all chromosomes have achieved bi-orientation, at which point the SAC becomes “satisfied” (it subsides). **(B)** In this model of Aurora B function, any kinetochore–microtubule interaction, even if erroneous, satisfies the SAC. Aurora B is not a SAC component, but its ability to recognize and correct improper attachment makes it activate the SAC indirectly through generation of unattached kinetochores (as an intermediate in error correction), which are considered the only source of SAC signal. **(C)** In this alternative model, any tensionless kinetochore is a source of SAC signal, albeit of different signal strengths (size of the red flashes). Aurora B is directly required both for error correction and for the SAC. **(B,C)** were adapted from Ref. (48).

Similarly to the EC, the SAC also requires kinetochores (42, 43). In contrast to the EC, however, the SAC has the ability to extend into a “global” signal that diffuses away from kinetochores and prevents mitotic exit in the presence of even a single unattached or improperly attached kinetochore (44). The SAC pathway converges on the assembly of a checkpoint effector complex, the mitotic checkpoint complex (MCC), which targets and inhibits the anaphase-promoting complex or cyclosome (APC/C, Figure 2A). The activity of this ubiquitin (Ub) ligase targets Cyclin B and Securin, which are, respectively, the activator of the main mitotic “engine”, the Cdk1 kinase, and a stoichiometric inhibitor of the protease Separase, which is required for dissolution of sister chromatid cohesion. Proteasome-dependent destruction of Cyclin B and Securin upon their ubiquitination by the APC/C inactivates Cdk1 and activates Separase, respectively, triggering mitotic exit and sister chromatid separation (Figure 2A) (4, 45). Cells in which the checkpoint is altered or artificially inactivated undergo precocious mitotic exit in the presence of unattached or incorrectly attached chromosomes and are therefore prone to mis-segregation events (44).

The general role of Aurora B activity in the EC and the SAC has been widely debated (28, 49). Early models based on experiments with attenuated alleles of Aurora B or at non-saturating doses of small-molecule inhibitors identified in Aurora B an exclusive component of the EC machinery (Figure 2B). According to these models, Aurora B contributed indirectly to SAC activation by generating unattached kinetochores, which became identified as the only structures capable of activating the SAC (32, 50).

This view has been progressively revised, partly because the molecular evidence in favor of a direct role of Aurora B in SAC control has been growing (34–36, 48, 51–54) and partly because there has been a conceptual evolution regarding what the SAC may be monitoring at kinetochores, with a shift from a pure “microtubule occupancy” model to an “intra-kinetochore tension” model (41, 55–58) (Figure 2C). Importantly, results obtained with different experimental approaches have caused the community to oscillate in their preference for a model or the other. However, a full assessment of the virtues and shortcomings of these models remains out of reach, as the molecular understanding of the conditions that lead to EC and SAC activation or silencing remains rudimentary, at least in relation to the considerable complexity of the process. Furthermore, while these two pathways are separable in their downstream components, they may be largely or even completely non-separable in the sensory apparatus that activates or switches them off at the “outer kinetochore,” where they operate. For instance, *cyclin-dependent kinase 1* (Cdk1), Aurora B, *monopolar spindle 1* (Mps1), and *budding uninhibited by benzimidazoles 1* (Bub1), all mitotic protein kinases, are required to promote correct kinetochore–microtubule-binding configurations, as well as for the SAC response (59). They are likely to regulate both phenomena at the same time and from the same place, the kinetochores. In this review, we focus on some of the molecular details that implicate Aurora B in these two pathways.

## Aurora B is a Subunit of the CPC

**Figure 3:**
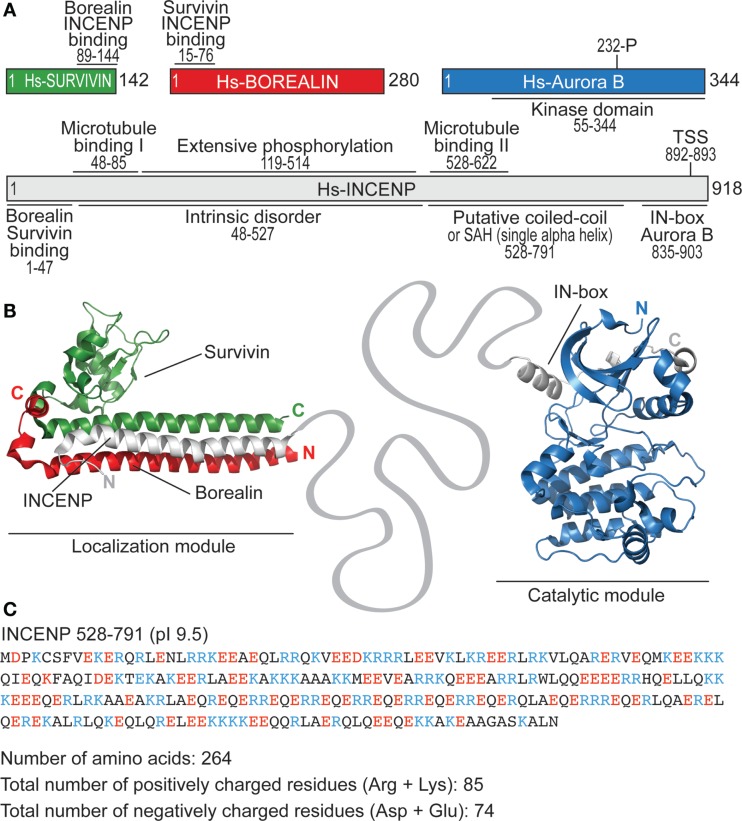
**Structural organization of the CPC**. **(A)** Schematic representation of human CPC subunits with main structural features. **(B)** Structural organization of the localization module of the CPC (PDB ID 2QFA) (77) and of the catalytic module (PDB ID 2BFX) (70). The linker between the N- and C-terminal regions of INCENP encompasses more than 800 residues. **(C)** Sequence of the putative coiled-coil region of INCENP shows that its features are hardly compatible with coiled-coil folding (due to insufficient number and irregular spacing of hydrophobic residues). pI defines isoelectric point.

Aurora B kinase is embedded in a multi-protein complex known as the chromosome passenger complex (CPC), whose subunits are codependent for stability and localization (60–64). The three additional CPC subunits are named inner centromeric protein (INCENP, and known as Sli15p in yeast)], Survivin (Bir1p) and Borealin (also known as CSC-1, Dasra, and Nbl1p) (63, 65–69) (Figure 3A). The CPC consists of two functionally distinct modules (20): a module delivering the catalytic activity, composed of Aurora B and a ~50-residue segment at the C-terminal end of INCENP, the so-called IN-box (62, 67, 70–74); and a module mediating localization, consisting of a ~45-residue segment at the N-terminal end of INCENP, Survivin, and Borealin (64, 75–84). The two modules are connected by the central part of INCENP (Figure 3B and see below).

Activation of Aurora B kinase arises from multiple regulatory steps, including the binding of the IN-box of INCENP around the Aurora B active site, the Aurora B-mediated phosphorylation of the IN-box on a Thr-Ser-Ser (TSS) motif, and the auto-phosphorylation of Aurora B at Thr232 (abbreviated as AB-T232-P) in the activation segment (70, 72, 85). Thus, Aurora B activation resembles that of many other kinases, in that it requires interaction with a partner protein and phosphorylation. Phosphorylation at the TSS and at the Aurora B activation segment is likely to occur in trans (70) and may therefore be sensitive to the local concentration of the CPC (86, 87).

Besides the intrinsic mechanisms of regulation described above, Aurora B may also be controlled by extrinsic mechanisms. For instance, phosphorylation of Ser311 of Aurora B by checkpoint kinase 1 (Chk1) may promote catalytic activation of Aurora B near kinetochores (88, 89). In addition, phosphorylation of the CPC targeting subunit Borealin by Mps1 has also been proposed to regulate Aurora B activity (90), but this remains controversial as neither Aurora B nor its activity are grossly perturbed by Mps1 inhibition (91–93). Furthermore, the protein TD-60 has been indicated as an additional CPC subunit required for CPC centromere targeting and Aurora B activation (94, 95). A recent study revealed that TD-60 is as a guanine nucleotide exchange factor (GEF) for the small Ras-like GTPase RalA, and that the latter modulates Aurora B activity and localization (96). While the mechanisms through which the RalA GEF activity of TD-60 influences Aurora B localization and activity requires further investigation, it seems now clear that TD-60 is not part of the CPC.

One of the most exciting chapters in the study of Aurora B kinase has been the development of highly specific and selective chemical inhibitors, spurred by the identification of this kinase as a potential target in oncology (97). Leaving clinical implications aside (98), small-molecule ATP-competitive inhibitors such as Hesperadin and ZM447439 proved invaluable tools for acute mitotic inhibition of Aurora B function and for the investigation of its mitotic functions in basic research laboratories (34, 35).

## The Localization Module of the CPC: Centromere Localization and Beyond

**Figure 4:**
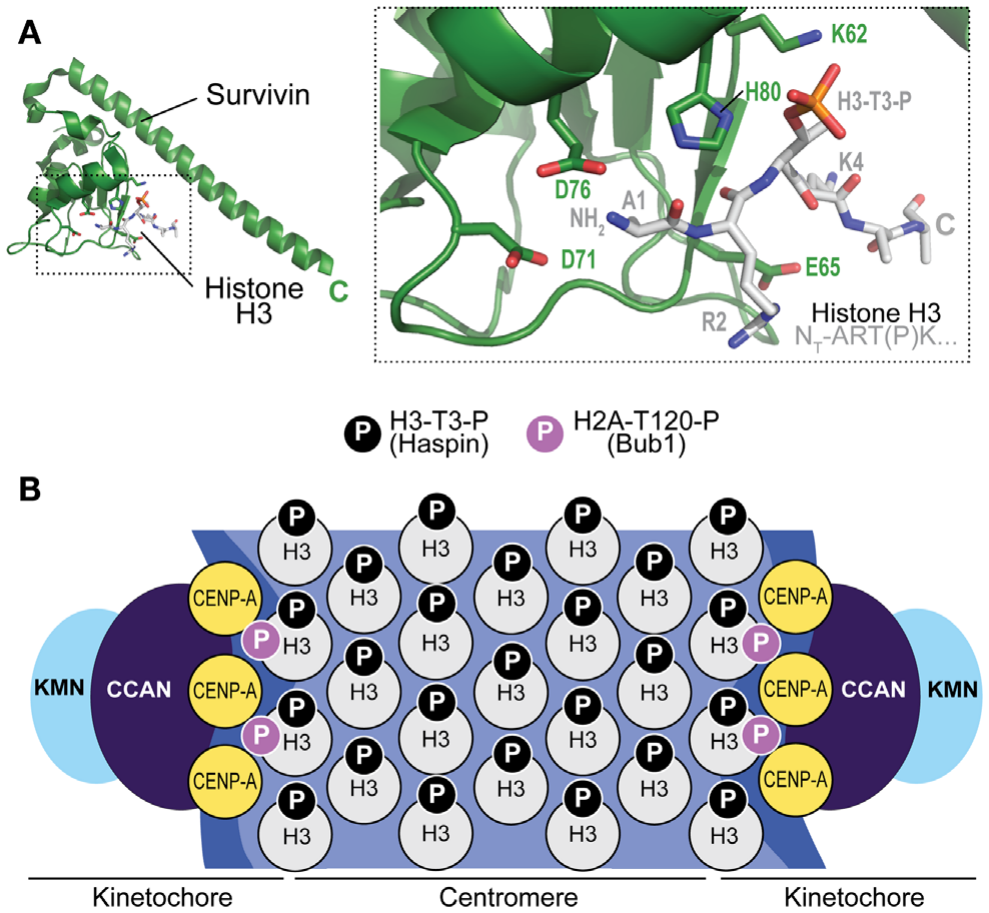
**Mechanism of CPC recruitment to centromeres**. **(A)** Crystal structure of the complex of Survivin with a peptide encompassing the N-terminal region of Histone H3 (PDB ID 4A0J). The peptide has sequence Ala-Arg-Thr(P)-Lys, where (P) indicates that Thr3 is phosphorylated. Asp71 (D71) is implicated in the recognition of the free N-terminus of Ala2 (the N-terminal Met1 is removed by an aminopeptidase). **(B)** Haspin kinase phosphorylates Thr3 of histone H3 (H3-T3-P) in the centromere region to allow recruitment of the CPC. Bub1 kinase phosphorylates Histone H2A on Thr120 (H2A-T120-P) near kinetochores (i.e., the modification does not extend to centromeres). In principle, both H3-containing and CENP-A containing nucleosomes may contain this modification.

The localization module targets the CPC to the centromere, where the bulk of the CPC localizes during mitosis. Crucial for centromere targeting is the CPC subunit Survivin, a member of a family of inhibitor of apoptosis (IAP) proteins containing a BIR domain (99). While Survivin might have lost its function as inhibitor of apoptosis, typical of other IAPs, its BIR domain has retained the ability of recognizing the N-terminus of target proteins. In Survivin, this ability is leveraged to bind a short N-terminal segment of Histone H3 (78, 80, 81) (Figure 4A). In fact, Survivin binds a short N-terminal segment of Histone H3 that must include a phosphorylated version of Thr3 (H3-T3-P) for efficient recognition (76, 78, 80, 81, 83, 84). The kinase responsible for this preeminently mitotic modification of Histone H3 is named Haspin (100). By recognizing H3-T3-P at centromeres, Survivin targets the CPC to the centromere (Figure 4B). Whether this H3-T3-P-dependent mechanism operates in yeast has remained unclear, because deletion of the yeast haspin-like kinases does not result in a growth defect phenotype (101). In yeast, a survivin-dependent mechanism may rely on the interaction of the Survivin homolog Bir1 with Ndc10, a subunit of the CBF3 centromeric complex (102, 103).

Besides H3-T3-P, also the phosphorylation of Thr120 of Histone H2A by Bub1 kinase (H2A-T120-P, H2A-S121-P in fission yeast) has been implicated in centromere recruitment of the CPC (81, 104, 105) (Figure 4B). The role of this mark, which is detected at kinetochores but not at centromeres, is more elusive. It appears to be crucial to regulate a homeostatic circuit that dynamically controls the activity and abundance of protein kinases, including Plk1 and Aurora B, and protein phosphatases, including members of the protein phosphatase 2A (PP2A) family associated with the B56 regulatory subunit (PP2A-B56), at kinetochores and centromeres. Specifically, H2A-T120-P is believed to promote recruitment of Shugoshin proteins (SGOL1 and SGOL2/TRIPIN in humans) (106, 107). These, in turn, control the recruitment of proteins that play a prominent role in error correction, including kinesin-13 family members such as MCAK, a microtubule depolymerase and Aurora B substrate, and the PP2A-B56 protein phosphatase complex, which balances abundance and activity of Aurora B and Plk1, as well as the phosphorylation of important CPC targets (108–118).

In addition to the recognition of histone marks, other mechanisms have been implicated in CPC centromere recruitment or activation, such as post-translational modifications of Survivin, Borealin, and INCENP, including phosphorylation by Aurora B itself, Cdk1, Mps1, and Plk1 (90, 119–123). Direct binding of Borealin to double-strand DNA has also been reported (64). Finally, there is evidence that oligomerization of the localization module also contributes to its localization (64, 124). How these features may impinge on CPC centromere targeting remains poorly understood.

## Incenp, the Bridge Connecting the Two CPC Modules

The functional properties of INCENP that have remained mechanistically obscure are now starting to emerge. INCENP is a rather large protein (918 residues for Isoform 1 in humans; source Uniprot: http://www.uniprot.org) (Figure 3A). Large parts of the INCENP primary sequence are low-complexity and unlikely to adopt a defined three-dimensional tertiary (and even secondary) structure. A predicted coiled-coil between residues 528 and 791 of INCENP is often considered an exception. More careful scrutiny, however, leads to exclude that this segment of INCENP is a genuine coiled-coil. It contains too few hydrophobic residues to support coiled-coil oligomerization and its frequent stretches of positively and negatively charged residues (Figure 3C) may produce false-positive classifications as coiled-coils. Analysis of INCENP residues 528–791 with REPPER, a program that detects short repeats and predicts periodicities in protein sequences (125), suggests that it lacks the regular sequence pattern typical of coiled-coils. In agreement with this idea, a very recent study on avian INCENP showed that this region (residues 503–715, corresponding to residues 528–791 of human INCENP in Figure 3A) folds as a single alpha helix (SAH) domain, rather than as a coiled-coil (126). SAH can unfold reversibly under force, thus extending up to 2.5-fold over their rest length. Because the N-terminal part of the SAH domain contains a second microtubule-binding domain (126), in addition to the one already identified in the N-terminus of INCENP (residues 48–85 of the human protein) (60, 74, 127, 128), it is a potential candidate for regulation by microtubule attachment.

INCENP also contains a large disordered region (residues 48–527, Figure 3A), stuffed with phosphorylation sites. The UNIPROT (http://www.uniprot.org) reports at least 24 phosphorylation sites in residues 119–514 of human INCENP. Phosphorylation of Thr388 in this segment has been implicated in binding and targeting of Plk1 (129). As discussed more thoroughly below, Cdk1-dependent phosphorylation of Thr59 within this segment has important consequences for CPC localization.

## The Spatial Separation Model of Aurora B Function

**Figure 5:**
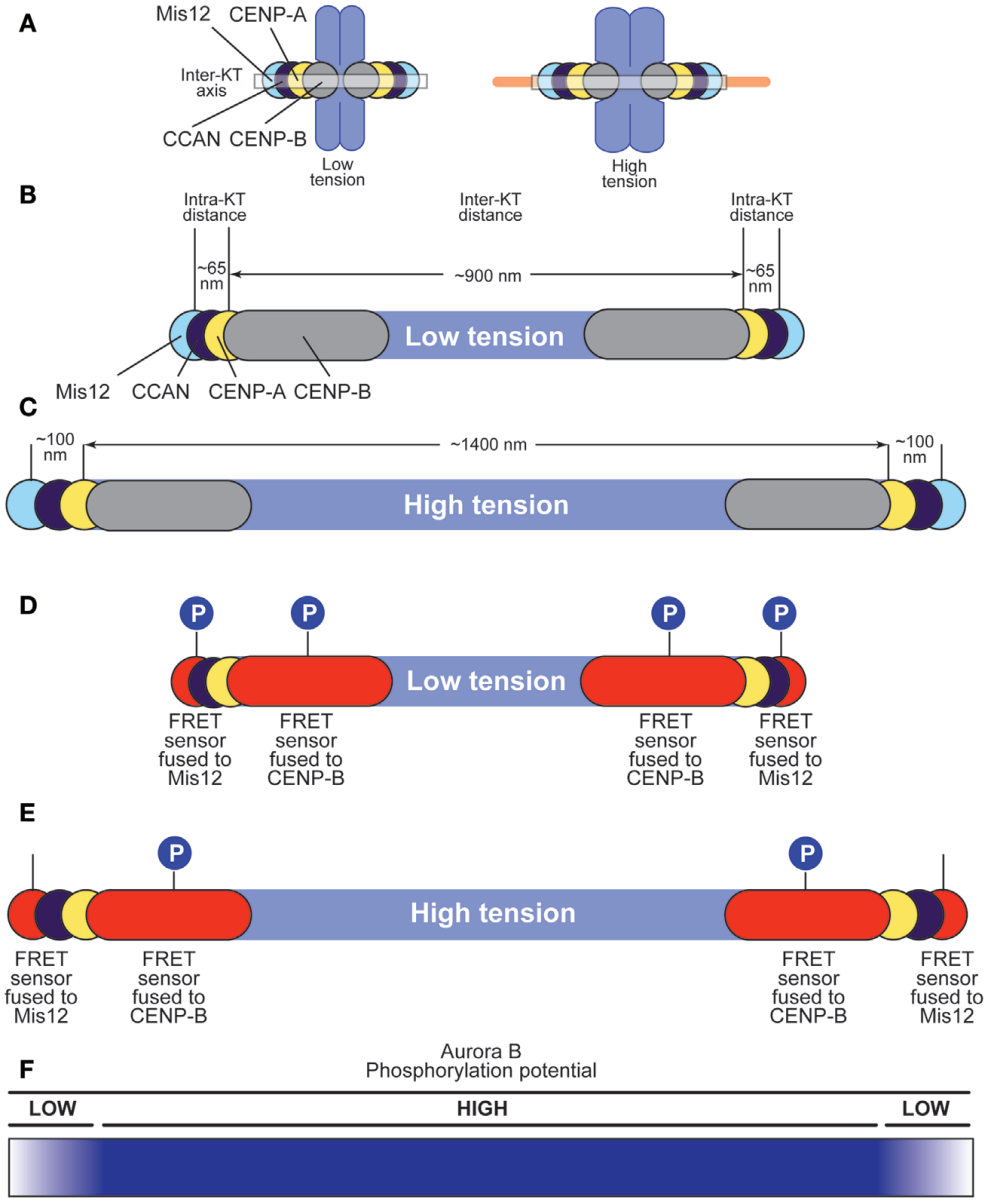
**Effects of tension on centromere and kinetochore structure**. **(A)** Chromosome lacking tension (left) or under tension (right). The centroid of the distributions of proteins in the kinetochore, including CENP-B, CENP-A, CCAN subunits, and Mis12 (part of the KMN network) is represented as a circle along the inter-kinetochore axis. Each centroid has a defined coordinate along the axis (57). Microtubules cause changes in the position of the centroids. **(B,C)** Under low tension **(B)**, inter-kinetochore distance in HeLa cells is ~900 nm (0.9 μm), whereas the distance between the centroids of the distributions of CENP-A and Mis12 is ~65 nm in *Drosophila* and as little as ~40 nm in human kinetochores (132). CENP-B binds the CENP-B box in alpha-satellite DNA at centromeres, and extends slightly beyond CENP-A toward the centromere (55). Under high tension **(C)**, inter-kinetochore distance grows to 1400 nm (1.4 μm), whereas the distance between the centroids of the CENP-A and Mis12 distributions grows to 100 nm (57). **(D,E)** A FRET sensor responding to Aurora B phosphorylation was fused either to CENP-B or to Mis12 (55). Under low tension **(D)**, the sensor is phosphorylated regardless of its position, suggesting that Aurora B can reach both positions with similar efficiency. Under high tension **(E)**, the outermost sensor cannot be phosphorylated efficiently (possibly because it becomes dephosphorylated), whereas the innermost sensor continues to be phosphorylated. **(F)** The phosphorylation potential of Aurora B decays very rapidly after the position defined by the innermost FRET sensor (fused to CENP-B) when chromosomes are under stretch. This rapid decay takes place in ~200 nm or less along the inter-kinetochore axis.

While it is clear that Aurora B substrates at centromeres and kinetochores undergo dynamic changes in their phosphorylation state during the relatively short time it takes kinetochores to attach to the spindle, it is uncertain to which extent these changes reflect the dynamic regulation of Aurora B activity by the intrinsic and extrinsic mechanisms discussed above (41). Rather, current models of Aurora B function focus primarily on the tension-dependent separation of Aurora B from its substrates as the basis of Aurora B regulation in EC and the SAC (41, 58). To appreciate this argument, it is important to explain the geometry of centromeres and kinetochores, their variation during bi-orientation, and how Aurora B may position itself within this system. The size of kinetochores is roughly equivalent to the wavelength of visible light, and in first approximation kinetochores appear as diffraction limited “spots” in the light microscope. In HeLa cells, the distance between the centroids of “spots” corresponding to inner kinetochore proteins in the two sister kinetochores (inter-kinetochore distance) grows from ~0.9 μm in the absence of microtubule binding in prometaphase (i.e., in the absence of tension) to ~1.4 μm upon bi-orientation at metaphase (when chromosomes are end-on-attached and under full tension) (130) (Figure 5A). Similar increases in inter-kinetochore distance have been measured in other cell types: inter-kinetochore distance increases from ~1.1 μm in the absence of microtubule binding to ~2.2 μm upon bi-orientation in newt lung cells (131), and from 0.72 to 0.94 μm in *Drosophila* S2 cells (57).

Thus, tension introduces macroscopic changes in the organization of the inter-kinetochore space between sister kinetochores. Importantly, tension also modifies the internal structure of the kinetochore, a condition referred to as intra-kinetochore stretch (Figure 5A). In S2 cells, for instance, the span of the kinetochore [from CENP-A to the centromere-proximal end of the Ndc80 subunit (also known as Hec1), measured along the inter-kinetochore axis] is ~65 nm in the absence of tension, and 102 nm in the presence of tension (57) (Figures 5B,C). Similar tension-driven increases in stretch are observed within human and yeast kinetochores (132, 133). The precise structural changes underlying the establishment of intra-kinetochore tension, however, remain unknown.

The spatial separation model builds on the observation that an Aurora B FRET sensor shows constitutive, tension-independent phosphorylation when positioned close to Aurora B at the interface between the centromere and inner kinetochore, but tension-sensitive phosphorylation when positioned more distantly from the kinase (55) (Figures 5D,E). More specifically, Aurora B phosphorylates a FRET sensor located at the centromere–kinetochore interface (because fused to the CENP-B protein) regardless of attachment status and despite the very significant increase in inter-kinetochore distance upon bi-orientation. Conversely, Aurora B phosphorylates the same FRET sensor now fused to a subunit of the Mis12 complex, in the outer kinetochore, when chromosomes are not under tension, but does so less effectively when tension is present at metaphase (55). Analogous observations have been made with *bona fide* Aurora B substrates (41, 55–58, 130, 134) and Aurora B substrates located in the outer kinetochore become progressively dephosphorylated during the attachment process (134–136). Furthermore, artificial repositioning of Aurora B to the outer kinetochore prevents dephosphorylation of outer kinetochore substrates (55). The persistence of Aurora B phosphorylation on a sub-class of “proximal” substrates despite full tension suggests that the kinase activity of Aurora B may not *per se* be force dependent.

Thus, it appears that certain substrates, and in particular substrates in the KMN network that mediates the EC and SAC responses, become physically separated from the kinase as tension arises (41). In the absence of tension, such as in syntelic attachment, substrates remain phosphorylated and attachments intrinsically unstable. In conclusion, the “phosphorylation potential” of Aurora B is dampened with a sharp edge within the very short distance that separates centromeres from kinetochores under tension, whereas it is largely insensitive to the considerable degree of stretching of the inter-kinetochore region (Figure 5F). This clearly suggests that Aurora B is able to read intra-kinetochore tension rather than inter-kinetochore tension, but how it achieves this has remained unclear.

## Reading Intra-Kinetochore Tension: From Centromeres?

It was initially hypothesized that the spatial separation that promotes stabilization of kinetochore–microtubule attachment might be linked to the increase in the distance from centromeres, where the bulk of Aurora B is positioned, to kinetochores, where the substrates of Aurora B that mediate microtubule attachment are located (33). With increasing distances, indicative of end-on attachment, the ability of Aurora B at centromeres to reach its substrates would progressively decrease, allowing a progressive stabilization of the kinetochore–microtubule interface. Implicit in this model is the existence of a sharp and separable centromere–kinetochore boundary, but a precise definition of what this boundary looks like is missing. If we consider that CENP-A is embedded in centromeric chromatin containing abundant histone H3 nucleosomes, H3-T3-P is likely to extend to the immediate periphery of kinetochores, and there is no obvious reason why the CPC should not bind to these H3 nucleosomes. It is unknown whether these nucleosomes become separated from CENP-A containing nucleosomes when tension builds up following microtubule end-on attachment to kinetochores.

**Figure 6:**
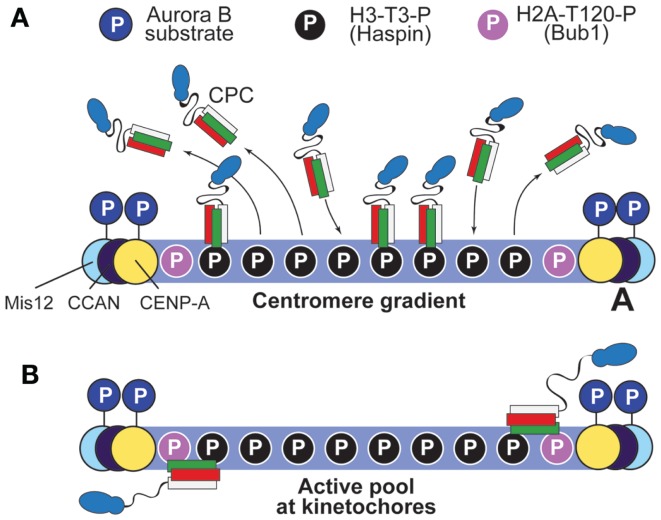
**Two possible models to account for the gradient of Aurora B phosphorylation**. **(A)** The “centromere gradient” model predicts that the CPC becomes recruited to centromeres as illustrated in Figure 4B, and it then dissociates from them, creating a gradient of CPC concentration (and therefore, by inference, of substrate phosphorylation). We note, however, that it is unlikely that this gradient could account for the sharp transition of phosphorylation potential of Aurora B within the very limited scale length of the kinetochore (see Figure 5). **(B)** An alternative model posits that an active form of the CPC is anchored near the kinetochore, and that the centromere pool is not strictly required for function (it was therefore omitted from the drawing). Proximity to H2A-T120-P might lead to the activation of this kinetochore pool of the CPC. Interactions with kinetochore subunits are also possible.

Because the subunits of the CPC turn over at centromeres with halftimes (*t*_1/2_) of <1 min (137–139), an alternative hypothesis is that a gradient of Aurora B substrate phosphorylation may be created by initial recruitment of the CPC to H3-T3-P to the centromere and by subsequent release and diffusion from centromeres (41) (Figure 6A). Indeed, this mechanism (“centromere gradient”) can create gradients of Aurora B substrate phosphorylation (140–142). We note, however, that such gradients are relatively flat and form over length scales of several micrometers (140–142). It is therefore unlikely that this diffusible gradient of Aurora B would generate the very sharp edge of activity observed within the ~100 nm (0.1 μm) length scale of the kinetochore.

The idea that the phosphorylation gradient of Aurora B is created by centromere recruitment and release of the CPC is also at odds with the results from experiments in which centromere enrichment of the CPC was prevented by targeted mutations in CPC subunits. For example, a Survivin mutant impaired in its ability to bind H3-T3-P supports chromosome segregation and long-term viability of DT40 cells deprived of endogenous Survivin (82). Similarly, the deletion of residues 1–228 of Sli1 (Sli15ΔN) rescues the lethality of *BIR1* in *S. cerevisiae*, even if Sli15ΔN does not localize to centromeres (see below) (101). Furthermore, phosphorylation of an inner kinetochore substrate of Aurora B, Ser7 of CENP-A (CENP-A-S7-P) is unaltered after depletion or inhibition of Haspin with 1 μM 5-ITu (5-iodotubercidin), a concentration of the drug that clears centromeres of H3-T3-P (78, 143). Altogether, these observations suggest that a gradient of Aurora B substrate phosphorylation at the centromere–kinetochore interface can be established also in the absence of Aurora B at centromeres.

## … Or from Kinetochores?

An alternative hypothesis for Aurora B function is that the functionally relevant pool of Aurora B resides near or at kinetochores, rather than centromeres (22, 59). Strikingly, it was shown that centromeric accumulation of Aurora B is subordinate to kinetochore establishment. An ectopic kinetochore built at a chromosome site containing a Lac-O array, by tethering segments of the kinetochore CCAN subunits CENP-C or CENP-T, promotes accumulation of H3-T3-P and of the CPC in an area comprised between the two ectopic tethering sites, suggesting that the ectopic kinetochore dictates the position of the “centromere” (144). Remarkably, the CENP-C and CENP-T segments used in these experiments do not recruit CENP-A, suggesting that the latter is not required for CPC recruitment at the ectopic “centromere.” Establishment of this ectopic centromere likely involves kinetochore-associated Bub1, which may promote the recruitment of Sgo1. Sgo1, in turn, plays a crucial role in the establishment and protection of centromeric cohesion (104, 107, 115, 145). In *S. cerevisiae*, the core centromere (a ~125 bp segment on which the kinetochore is built) and two kinetochore proteins, Iml1 and Chl4 (respectively, related to the CCAN subunits CENP-L and CENP-N in humans), are important for the spreading of Sgo1 to pericentromeric regions (146).

Further emphasizing the role of kinetochores in CPC localization is the observation that the abundance of the centromere pool of Aurora B in diploid human cells is controlled dynamically by kinetochore attachment status, with misaligned chromosomes showing an enrichment of Aurora B (135). En passing, this dynamic kinetochore-driven enrichment of Aurora B at centromeres requires Aurora B and Plk1 activity, but not Mps1’s (135). The dispensability of Mps1 is further testified by experiments showing that chemical inhibition of Mps1 activity does not affect the total levels of H3-T3-P, the phosphomark that recruits the CPC to centromeres (124). However, Mps1 may modulate the timing of CPC accumulation at the centromere (147).

The role of kinetochores is further supported by the observation that Knl1, one of the outer kinetochore KMN subunits, is required for Aurora B activation, and that the active form of Aurora B (monitored through AB-T232-P) resides at kinetochores rather than at centromeres (148–150). Identifying the precise reason for this is a crucial question for future analyses. It is plausible that the centromere, contrary to the prometaphase kinetochore, represents a domain of high phosphatase activity that prevents the accumulation of the active form of Aurora B. The kinetochore pool of Aurora B was recently observed under conditions of Haspin inhibition, and was shown to depend on dimerization of Borealin (124).

Sli15ΔN, the Sli15 mutant discussed in the previous section, is also observed at kinetochores in cells depleted of Bir1/Survivin, suggesting that kinetochore localization of this mutant is survivin independent (101). Remarkably, while the Sli15ΔN mutant rescued chromosome segregation and the lethality associated with loss of Bir1/Survivin in *S. cerevisiae*, it was synthetic lethal with two normally non-essential CCAN subunits at the kinetochore, Mcm21 and Ctf19 (respectively, homologous to CENP-O and CENP-P of higher eukaryotes) (101, 151). These two proteins and their binding partners Okp1 and Ame1 (considered to be homologous to CENP-Q and CENP-U, respectively) have been previously shown to promote kinetochore recruitment of the CPC and to be necessary for the error correction activity of the CPC in *S. cerevisiae* (152–154).

How Aurora B is recruited to kinetochores is unclear, but a hint comes from the observation that INCENP is phosphorylated on Thr59 (INC-T59-P) by Cdk1 kinase, the master regulator of cell division (64, 129, 155). A phosphomimetic T59E mutant causes INCENP to persist on chromosomes rather than to become relocated on the central spindle at anaphase (155, 156). With the decline in tension upon sister chromatid dissolution in anaphase, the T59E mutant causes the re-activation of typical Aurora B-dependent events at kinetochores, including the recruitment of Mps1, Bub1, and BubR1. Thus, “stripping” of the CPC from centromeres might be required to prevent EC and SAC re-activation during anaphase. However, retention of CPC localization is *per se* not sufficient for a complete re-activation of these pathways (156–160).

Of note, both H3-T3-P and H2A-T120-P are removed from centromeres at anaphase (81, 161), suggesting that the T59E INCENP mutant may not be retained on chromosomes through these phosphomarks but via a different, currently uncharacterized interaction. Phosphorylation of INC-T59-P may prevent an interaction of INCENP with the MKLP2 kinesin, which is required to relocate the CPC to the central spindle at anaphase (156, 162, 163). Alternatively, it might mediate a direct interaction with one or more kinetochore subunits. This pathway is conserved in *S. cerevisiae*, where dephosphorylation of Cdk1-dependent sites on Sli15 was shown to be important for CPC relocation at anaphase (123).

**Figure 7:**
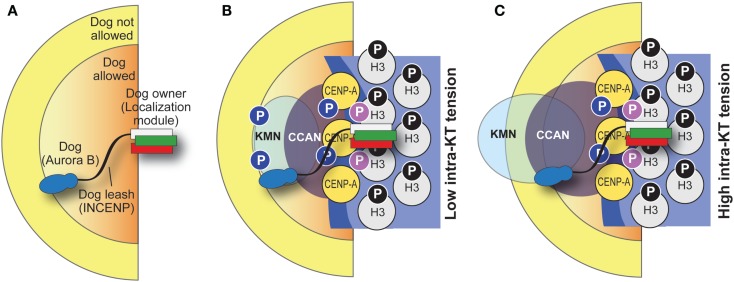
**“Dog leash” model of CPC function**. **(A)** The idea behind the “dog leash” model is that the localization module of the CPC (the owner) is tethered at the base of kinetochores. INCENP acts as a “dog leash” that allows the “dog,” Aurora B, to phosphorylate substrates only within limits defined by the length of the linker (which may vary, e.g., as a consequence of phosphorylation). This defines a boundary between regions where the dog is allowed and regions where it is not. **(B,C)** Application of the dog leash model to kinetochores. Under low tension **(B)**, Aurora B can reach out in the kinetochore and phosphorylate substrates there. Under high tension **(C)**, substrates (e.g., in the KMN network) have crossed the boundary defined by the leash and become unreachable. Note that in this drawing the CPC is tethered at the base of the kinetochore and its position is stationary, but this may not be the case and tension might increase its distance from the CENP-A base of the kinetochore. The function of a phosphatase is implicit in the model.

Thus, the active CPC pool that generates the intra-kinetochore phosphorylation gradient discussed above may reside within kinetochores, rather than being delivered there by a diffusible gradient of the kinase (Figure 6B). How does this kinetochore pool of the CPC generate the observed phosphorylation gradient within kinetochores? We have previously proposed that INCENP might act as a flexible arm whose maximal extension limits the reach of Aurora B within kinetochores (22). In this “dog leash” model, intra-kinetochore stretch promoted by microtubule binding might create relative movements of the Aurora B substrates relative to the tethered CPC, until substrates become unreachable by the kinase (Figure 7). As discussed above, the coiled-coil domain of INCENP has been recently shown to contain a SAH domain (126). In agreement with the “dog leash” model, it was shown that the length of the SAH domain modulates the ability of Aurora B to reach its substrates in the centromere and in the outer kinetochore (126).

## … Or from Microtubules?

Yet, another hypothesis is that Aurora B performs its functions from microtubules (101). This theory builds on previous work characterizing INCENP/Sli15 as a microtubule-binding protein (discussed above). Indeed, Sli15ΔN, the already discussed deletion mutant rescuing the lethality of the *bir1* deletion in *S. cerevisiae*, localizes strongly to microtubules (101). Another recent study suggests that microtubules regulate Aurora B localization and activity in prometaphase (164). Albeit attractive, the hypothesis that microtubule localization is sufficient for CPC function requires further evaluation, not least because CPC function is delivered also in cells lacking microtubules altogether (e.g., because treated with spindle poisons). We reason that because the Sli15ΔN mutant retains kinetochores localization, the most parsimonious interpretation of its ability to suppress the lethality of the *bir1* deletion is that it does so from kinetochores.

## A Model for CPC Localization and Function

**Figure 8:**
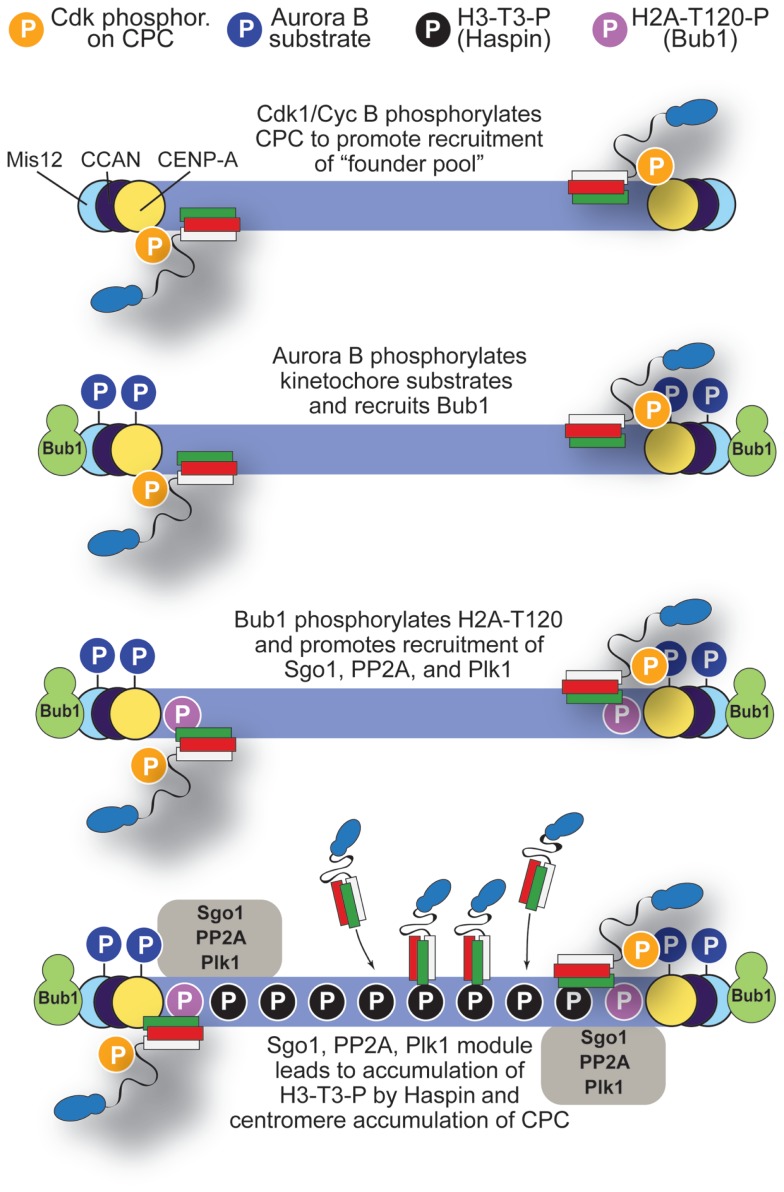
**A model for localization of CPC**. The initial step in accumulation of CPC to centromeres may involve Cdk1/Cyclin B, which phosphorylates INCENP-T59 and possibly other sites on CPC. Initial CPC activity at kinetochores recruits Bub1, which in turn, after phosphorylating H2A-T120, promotes recruitment of Sgo1, PP2A, and Plk1. These, in turn, promote activation of Haspin and centromere accumulation of the CPC. See main text for additional detail.

Based on the discussion above, we propose a tentative model for the mechanism of CPC localization (Figure 8). Cdk1-mediated phosphorylation of INCENP may be the initial trigger causing the recruitment of a pool of the CPC to kinetochores through interactions with yet to be identified subunits, possibly within the CCAN at the inner kinetochore. At kinetochores, Aurora B contributes to the recruitment of Bub1 kinase, which creates H2A-T120-P to recruit a kinetochore pool of Sgo1 (107, 115). The latter is responsible for the homeostatic control of phosphorylation at kinetochores through recruitment of PP2A phosphatase and Polo-like kinase 1 (Plk1). How these proteins interact at kinetochores is largely unclear and requires further analysis.

We surmise that execution of this pathway may have two main consequences: (1) limiting the activation of Aurora B to the kinetochore pool and (2) igniting a positive feedback loop that promotes Haspin activation and further CPC accumulation at centromeres via phosphorylation of H3-T3-P in neighboring H3 nucleosomes. Aurora B itself, Plk1, Bub1, and, to a lesser extent, Mps1 may be involved in this positive feedback loop (62, 120, 121, 124, 135, 145, 147, 165). Although Bub1 acts downstream from Mps1 in the SAC pathway (3), there is significant residual Bub1 at kinetochores of cells in which Mps1 activity has been inhibited (91, 92).

Of note, H2A-T120-P is limited to kinetochores (115). Although it has been proposed that the CPC may localize at the intersection of H2A-T120-P and H3-T3-P (81), the overlap between these two marks may be limited to the inner kinetochore, whereas the localization domain of Aurora B is broader and clearly extends to the centromere. However, H2A-T120-P may contribute, by recruiting Shugoshin, to limit the activation of Aurora B to the kinetochore pool (149), although the details of this mechanism remain obscure. Sgo1 may also provide another anchoring point for the CPC at kinetochores, as the BIR domain of Survivin recognizes the N-terminal region of Sgo1 (76).

## Mechanisms of Error Correction

A comprehensive picture of the contribution of Aurora B to the establishment of bi-orientation is still missing, but there has been substantial progress in recent years. Importantly, Aurora B has also been shown to regulate the structural stability of the kinetochore. For instance, it phosphorylates the CCAN subunit CENP-C/Mif2 to confer robustness to kinetochore function (166). In addition, phosphorylation of human Dsn1/Mis13 at two closely spaced residues (S100 and S109) increases the binding affinity of the Mis12 complex for CENP-C (42, 134, 166–171).

As already discussed above, anaphase retention of the CPC on kinetochores by expression of the T59E INCENP mutant or by suppression of MKLP2 (see above) leads to loss of tension that re-activates Aurora B-dependent pathways, including re-recruitment of Mps1, Bub1, and BubR1 (156). Nevertheless, kinetochores remain attached to their microtubule fibers under these conditions, indicating that re-activation of Aurora B is not sufficient for error correction. The crucial missing factor is the activity of Cdk1, which declines at anaphase due to degradation of Cyclin B. Artificial retention of Cdk1 activity in cells that have undergone sister chromatid separation leads to extensive destabilization of kinetochore–microtubule ­attachments (158, 159, 172).

Aurora B contributes at least three, partly related functions, to the process of bi-orientation: (1) the modulation of microtubule-binding affinity of the kinetochore to allow or prevent maturation of attachments; (2) the regulation of microtubule dynamics by controlling the activity and localization of microtubule-associated proteins; (3) the control of the localization of additional proteins involved in the regulation of kinetochore–microtubule attachment, including protein phosphatases that antagonize the phosphorylation of Aurora B substrates (4, 173).

A widely studied example of how Aurora B modulates the affinity of kinetochores for microtubules is the phosphorylation of multiple residues on a disordered and positively charged ~80-residue tail at the N-terminus of Ndc80/Hec1, a subunit of the Ndc80 complex (30, 31, 174–178). This segment of Ndc80 neighbors a calponin-homology (CH) domain that binds directly to microtubules (174, 179). Different models have been proposed for how Ndc80 phosphorylation modulates the binding affinity of the Ndc80 complex for microtubules (174, 175, 178, 180–182). A rigorous recent analysis suggested that each new phosphorylation event on the Ndc80 tail determines a relatively small decrease in microtubule-binding affinity by the Ndc80 complex, regardless of which specific position, among the eight or nine available, becomes phosphorylated (177). In this model, the phosphorylation sites of the Ndc80 tail configure a “rheostat” capable of increasing the microtubule-binding affinity of individual Ndc80 complexes by a factor as small as 20- and as large as 100-fold when transiting from a fully phosphorylated form of the protein to a fully dephosphorylated one (174, 177). Because the degree of phosphorylation of the Ndc80 tail is maximal when tension is low (e.g., in the absence of microtubules) (148), it is plausible that dephosphorylation of the Ndc80 complex is a gradual process that occurs concomitantly with the generation of tension within kinetochores. Consistent with this hypothesis, expression of a non-phosphorylatable mutant of the Ndc80 complex leads to hyper-stretched kinetochore–microtubule attachment and frequent attachment errors (31, 148, 181). Ndc80 has also been shown to have a direct influence on the dynamics of kinetochore microtubules, and Ndc80 phosphorylation may influence this property (183). Importantly, another Aurora family member, Aurora A, has also been very recently implicated in this correction mechanism (184, 185).

In addition to microtubule binding by the KMN network, other Aurora B substrates are important for the stabilization of the kinetochore–microtubule interface. The Dam1 complex in *S. cerevisiae* and the SKA complex in higher eukaryotes are structurally unrelated but may perform analogous functions as stabilizers of kinetochore–microtubule attachment (186–193). Contrary to the Ndc80 complex, both the Dam1 and the SKA complexes are able to form processive, load-bearing attachments to depolymerizing microtubule *in vitro*, and both contribute to retaining the Ndc80 complex at depolymerizing microtubule tips, possibly enhancing the overall processivity of microtubule binding (191–197). Importantly, Aurora B phosphorylation negatively regulates the association of the SKA and Dam1 complexes with Ndc80 (194–196, 198–201). An analogous pattern is also observed for the kinetochore recruitment of another microtubule-binding complex, the Astrin–SKAP complex (202). Thus, recruitment of these additional microtubule-binding complexes likely “seals” the kinetochore–microtubule interface of bi-oriented sister chromatids on which the phosphorylation of Aurora B has already faded.

Aurora B also controls kinetochore localization and activity of the non-conventional kinesin-13 family member mitotic centromere-associated kinesin (MCAK, Kif2C), which plays an important role in error correction as a microtubule depolymerase at microtubule ends (39, 203–210). Kinetochore and centromere recruitment of MCAK requires Aurora B phosphorylation of MCAK (203, 206, 210) and the presence of Sgo2 (113, 114, 118, 211).

Also dependent on Aurora B is the recruitment of CENP-E, a kinesin that plays an important role in the initial, lateral attachment of kinetochores to microtubules that precedes end-on attachment (7, 35, 212, 213). Conversion from an initial lateral attachment to end-on attachment occurs also in budding yeast (214). It has been proposed that lateral attachments may be insensitive to Aurora B activity, and therefore may be able to provide a mechanism for establishment of initial kinetochore–microtubule attachments even when Aurora B activity is high (4, 200).

Finally, Aurora B is in an antagonistic relationship with protein phosphatases, most notably of the protein phosphatase 1 (PP1) and PP2A-B56 families (4). These phosphatases counter phosphorylation by Aurora B kinase and other downstream kinases both in the EC and in the SAC (15, 109, 215–218). Many details of the complex molecular mechanisms subtending to the antagonism of Aurora B and PP1 and PP2A phosphatases remain to be elucidated. The following examples illustrate the complexity of this regulation.

Distinct interactions of the B56 regulators with Sgo1, Sgo2, and with the checkpoint component BubR1 recruit the PP2A holoenzyme to centromeres and kinetochores during mitosis (108, 115, 211, 219). The interaction of PP2A-B56 with BubR1 requires the so-called kinetochore attachment regulatory domain (KARD) motif of BubR1, which undergoes multisite phosphorylation (presumably) at kinetochores, partly mediated by Plk1 (219). Interference with the interaction of PP2A with the KARD domain leads to an elevation of Aurora B substrate phosphorylation in the outer kinetochore and prevents the stabilization of kinetochore–microtubule attachment (219).

Repo-Man, a protein scaffold that interacts with the PP1 phosphatase, is responsible for the clearance of the Haspin-mediated phosphorylation of H3-T3-P (161). Aurora B counteracts the chromatin recruitment of Repo-Man by phosphorylating it on Ser893, thus ultimately preventing the dephosphorylation of H3-T3-P. Dephosphorylation of Ser893, which might follow the release of the CPC from its centromeric localization at anaphase, requires an interaction of Repo-Man with PP2A, which is mediated by a motif closely related to the KARD motif of BubR1 (220).

Kinetochore recruitment of PP1 requires interactions with Knl1 and with CENP-E (15, 16, 221). Aurora B prevents kinetochore targeting of PP1 by phosphorylating a PP1-docking motif on Knl1 (15, 16). In *S. cerevisiae*, a requirement for kinetochore recruitment of PP1 to Knl1 (known as Spc105 in this organism), without which the SAC cannot be silenced, resulting in cell lethality, can be bypassed if Aurora B activity is compromised (16). Both PP1 and PP2A have been implicated as suppressors of the Mps1-dependent phosphorylation of the multiple Met-­Glu-Leu-Thr (MELT) repeats of Knl1 that provide a docking site for the Bub1/Bub3 complex at kinetochores (215, 216).

## Aurora B in the SAC

The SAC effector MCC consists of three SAC proteins, Mad2 (mitotic arrest deficient 2), Bub3 (budding uninhibited by benzimidazoles 3), BubR1 (Bub1-related 1, the human ortholog of yeast Mad3), and the APC co-activator Cdc20. Additional SAC components are Mad1, the kinases Mps1 (monopolar spindle protein 1), and Bub1 (budding uninhibited by benzimidazoles 1), and, limitedly to metazoans, the components of the ­Rod-Zwilch-ZW10 complex (RZZ). All SAC components contribute to the formation of the MCC and therefore to APC/C inhibition (3, 45).

The mechanisms through which Aurora B regulates the SAC are likely to be closely interwoven with the mechanisms that trigger error correction. As already pointed out in the previous paragraph, retention of Aurora B activity on chromosomes during anaphase is insufficient to cause error correction, but is sufficient to recruit *bona fide* SAC components, such as Mps1, Bub1, and BubR1, despite the retention of robust kinetochore fibers (156, 158–160, 172). This observation argues that Aurora B plays a direct role in the recruitment of the SAC components also in the absence of error correction and of unattached kinetochores. Incidentally, the observation that Mps1 can be recruited to anaphase chromosomes that have retained kinetochore fibers needs to be reconciled with the recent proposition that microtubules compete directly with Mps1 localization to kinetochores (222, 223).

Aurora B appears to occupy an upstream position in the pathway of recruitment of SAC components, as its inhibition prevents kinetochore recruitment of all other SAC components (35, 48, 52, 224, 225). Co-inhibition of Aurora B and Mps1 has profound synergistic effects in the impairment of SAC signaling (48, 52). Aurora B inhibition prevents Mps1 recruitment, and artificially tethering Mps1 to the kinetochore bypasses the checkpoint requirement for Aurora B in human cells, suggesting that a primary function of Aurora B in the SAC is the recruitment of Mps1 (51, 52). Conversely, when a downstream SAC component, such as Mad1:Mad2 is tethered to kinetochores, the resulting mitotic arrest depends on Aurora B (51, 226, 227).

Mps1 becomes recruited to the Ndc80 complex of the kinetochore (222, 223, 228, 229). The precise role of Aurora B in the recruitment of Mps1 remains unclear, but a role of Ndc80 phosphorylation has been suggested (223, 229). However, the observation that Aurora B activity becomes at least partly dispensable for kinetochore recruitment of Mps1 when the Mps1 TPR region is deleted suggests that Aurora B does not need to generate a docking site for Mps1 on Ndc80 but rather regulates a conformational transition within Mps1 (230).

After its Aurora B-dependent recruitment to kinetochores, Mps1 promotes the recruitment of downstream SAC component by phosphorylating Knl1 on multiple MELT repeats to dock the Bub1:Bub3 complex (231–234). The latter, in turn, elicits the formation of a comprehensive assembly of SAC protein that may facilitate SAC signaling from kinetochores (235–240).

## Conclusion

Aurora B and the CPC are crucial for successful chromosome segregation during cell division. The two pathways Aurora B controls, error correction and the SAC, are tightly interwoven and interdependent. Both appear to rely on spatial control of Aurora B activity, but the precise molecular basis for this spatial control remains unknown. Future analyses will have to rigorously test the implications of the models that have been proposed to explain the spatial regulation of Aurora B activity, including the “centromere gradient” model and the “dog leash” model. It is hoped that global analyses of Aurora B substrate phosphorylation within the framework of predictable alterations of CPC and kinetochore function will finally shed light on the molecular basis of a mechanism that is indispensable for life.

## Conflict of Interest Statement

The authors declare that the research was conducted in the absence of any commercial or financial relationships that could be construed as a potential conflict of interest.
